# T-cell specific antibody induction versus corticosteroid induction immunosuppression for liver transplant recipients: a meta-analysis

**DOI:** 10.1038/s41598-023-32972-z

**Published:** 2023-04-28

**Authors:** Woo-Seok Jung, Jae Hee Kuh, Leerang Lim, Hae Kyung Yoo, Jae-Woo Ju, Ho-Jin Lee, Won Ho Kim

**Affiliations:** 1 Department of Anesthesiology and Pain Medicine, Seoul National University Hospital, Seoul, Republic of Korea; 2 Department of Anesthesiology and Pain Medicine, Seoul National University College of Medicine, 101 Daehak-ro, Jongno-gu, Seoul, 03080 Republic of Korea

**Keywords:** Hepatology, Outcomes research

## Abstract

Corticosteroids remain the mainstay of immunosuppression for liver transplant recipients despite several serious complications including infection, hepatitis C virus (HCV) recurrence, diabetes mellitus (DM), and hypertension. We attempted to compare the safety and efficacy of T-cell specific antibody induction with complete corticosteroid avoidance. We searched MEDLINE, EMBASE, and Cochrane central library. Randomized controlled trials comparing T-cell specific antibody induction with corticosteroid induction immunosuppression were included. Our primary outcome was the incidence of biopsy-proven acute rejection. Eleven trials involving 1683 patients were included. The incidence of acute rejection was not significantly different between the antibody and steroid induction groups (risk ratio [RR] 0.85, 95% confidence interval [CI] 0.72, 1.01, *P* = 0.06, I^2^ = 0%). However, T-cell specific antibody induction significantly reduced the risk of cytomegalovirus infection (RR 0.48, 95% CI 0.33, 0.70, *P* = 0.0002, I^2^ = 3%), HCV recurrence (RR 0.89, 95% CI 0.80, 0.99, *P* = 0.03, I^2^ = 0%), DM (RR 0.41, 95% CI 0.32, 0.54, *P* < 0.0001, I^2^ = 0%) and hypertension (RR 0.71, 95% CI 0.55, 0.90, *P* = 0.005, I^2^ = 35%). Trial sequential analysis for acute rejection showed that the cumulative z-curve did not cross the Trial sequential boundary and the required information size was not reached. T-cell specific antibody induction compared to corticosteroid induction seems to significantly reduce opportunistic infections including cytomegalovirus infection and HCV recurrence and metabolic complications including DM and hypertension. However, given the insufficient study power, low quality of evidence, and heterogeneous immunosuppressive regimens, our results should be cautiously appreciated.

## Introduction

Corticosteroids and calcineurin inhibitors remain a mainstay of immunosuppression in liver transplantation^1^. However, treatment with corticosteroids is associated with an increased risk of infection, diabetes mellitus (DM), obesity, and hypertension^2,3^. Thus, early withdrawal or complete avoidance of corticosteroids by adding another induction immunosuppressive agent such as T-cell specific antibody has been investigated^1,4,5^. However, whether corticosteroid minimization provides clear benefits remains unclear^3^.

T-cell specific antibody induction has been investigated to minimize corticosteroids in liver transplant recipients^1,6^. Induction immunosuppression using T-cell specific antibodies is now available and is used in approximately one-third of liver transplant recipients during the past decade^7^. T-cell specific antibodies used for induction immunosuppression include interleukin-2 receptor antagonists (daclizumab, or basiliximab), monoclonal antibodies specific for the CD3 receptor (muromonab-CD3) or the CD52 surface protein (alemtuzumab), and polyclonal antibodies (rabbit or horse anti-thymocyte globulin (ATG))^6^.

Four meta-analyses have been published regarding the use of T-cell specific antibody induction for complete avoidance of corticosteroids^8–11^. However, two meta-analyses included trials with different concomitant immunosuppression^8,9^, and one meta-analysis included non-randomized studies^11^. The previous meta-analysis including only randomized controlled trials with identical concomitant immunosuppression other than antibody and steroid showed that T-cell specific antibody induction significantly reduced DM and cytomegalovirus infection^10^. However, the quality of evidence was low for both outcomes due to the high risk of bias and imprecision^10^. Also, while trial sequential analysis (TSA) for diabetes mellitus revealed a significant benefit of T-cell antibody, TSA for cytomegalovirus infection did not. TSA for both outcomes showed that sufficient study power has not been reached^10^. Furthermore, as the included studies are heterogeneous in study protocols, higher quality evidence with additional randomized trials is required.

In this updated meta-analysis, we sought to compare the safety and efficacy of T-cell specific antibodies with corticosteroid induction in liver transplantation. We also performed subgroup analysis for different classes and types of antibodies and TSA to evaluate whether any class or drug type has a significant benefit over others.

## Methods

Our analysis protocol was registered on PROSPERO (registration number: CRD42022368664). We did not make any deviations from our pre-registered protocol. This meta-analysis was performed according to the Cochrane Handbook for Systematic Reviews of Interventions^12^. The meta-analysis results were reported according to the Preferred Reporting Items for Systematic Reviews and Meta-Analyses (PRISMA) statements^13^.

We searched EMBASE, MEDLINE, and the Cochrane Database of Systematic Reviews until October 22, 2022 from inception. We combined search terms for liver transplantation and T-cell specific antibodies. The search strategy of Medline was (Transplant* OR Graft*) AND (Liver OR Hepatic) AND (Antithymocyt* OR Thymoglobulin OR Monoclonal antibod* OR ATG OR ATGAM OR Thymus anti* OR Thymocyt* OR Muromonab OR OKT3 OR Orthoclone OR Simulect OR Basiliximab OR Daclizumab OR Dacluzumab OR Daclizimab OR Campath OR Alemtuzumab OR Zenapax). Only randomized trials written in the English language were considered.

We included randomized controlled trials comparing T-cell specific antibody induction to corticosteroid induction in patients undergoing liver transplantation of all ages. Patients with other transplanted organs, previous liver transplants, and ABO-incompatible liver transplants were excluded. Other concomitant immunosuppression regimens such as calcineurin inhibitors and/or mycophenolate mofetil were allowed if received equally by all intervention groups. Randomized controlled trials were restricted to those written in the English language. Two of our authors (WSJ and WHK) independently screened for and assessed eligible trials. The software used was Review Manager (RevMan version 5.4. Copenhagen: The Cochrane Collaboration, The Nordic Cochrane Center, Oxford, United Kingdom). No automation tool was used in the process.

Data including first author, country of origin, trial design, inclusion and exclusion criteria, sample size, induction immunosuppression regimens, maintenance immunosuppression regimens, and participant characteristics were collected by two authors (WSJ and JHK). Another author (WHK) confirmed the accuracy of collected data and any discrepancy was resolved by discussion.

The primary outcome was the incidence of biopsy-proven acute rejection. The secondary outcomes were patient safety-related outcomes including all-cause mortality, graft loss including patient death, acute rejection requiring treatment, corticosteroid resistant rejection, infection, CMV infection, hepatitis C virus (HCV) recurrence, malignancy, post-transplant lymphoproliferative disorder, the total length of hospital stay; renal function related outcomes including renal failure requiring dialysis, glomerular filtration rate, serum creatinine; and metabolic complications including diabetes mellitus, hypertension, hyperlipidemia, serum cholesterol.

After determining all included studies, the risk of bias of included trials was assessed using the Cochrane risk-of-bias tool version 2 for randomized clinical trials^12^. These following fields were assessed: bias due to deviations from the intended interventions, bias arising from the randomization process, bias in the measurement of the outcome, bias due to missing data, and bias in the selection of the reported result. Two authors independently assessed the risk of bias in included studies. We resolved disagreements by discussion by including, if necessary, a third author.

We evaluated the quality of the evidence for each of the outcomes according to the GRADE (Grading of Recommendations, Assessment, Development, and Evaluation) system. We used all five domains of GRADE: risk of bias, publication bias, indirectness, inconsistency, and imprecision^14^.

### Statistical analysis

Analyses were conducted using Review Manager (RevMan version 5.4. Copenhagen: The Nordic Cochrane Centre, The Cochrane Collaboration, Oxford, United Kingdom). We extracted mean and standard deviations for continuous variables. If continuous variables were reported as median and range, we assumed the mean to be equivalent to the median and estimated the standard deviation to be the range divided by four^12^. A fixed-effects model was adopted to calculate the effect size of our outcome variables. We used the inverse variance method for continuous outcomes and the Mantel–Haenszel method for dichotomous outcomes. The effect size was reported as a pooled mean difference (MD) or risk ratio (RR) with a 95% confidence interval (CI). Forest plots were depicted for acute rejection and new-onset DM. We assessed heterogeneity by the coefficient I^2^. Heterogeneity was graded by predetermined thresholds for high (more than 75%), moderate (50–74%), and low (less than 49%) levels^15,16^.

Publication bias was evaluated by visual examination of a funnel plot. Egger’s linear regression test and Duval and Tweedie’s trim and fill test were performed to assess the publication bias using STATA version 14.0 (standard edition, StataCorp, College Station, Texas, USA).

Meta-regression analyses were conducted including recipient age, sex ratio, laboratory MELD score, and cold ischemia time as moderators to explore potential sources of heterogeneity regarding the primary outcome (acute rejection).

To evaluate whether different immunosuppression regimens have a different impact on our outcomes, subgroup analyses were performed according to (1) different classes of antibodies (interleukin 2-receptor antagonists vs. polyclonal antibodies), (2) different formulations of the same class of antibody (basiliximab vs. daclizumab), and (3) the administration of an intraoperative corticosteroid bolus. Sensitivity analyses were conducted by analyzing our data using a random-effects model to calculate the effect size of our study outcomes.

A TSA was performed using TSA Viewer (Version 0.9.5.10 Beta, Copenhagen Trial Unit, 2016, Copenhagen, Denmark)^17^. A cumulative meta-analysis was performed using the cumulative number of events and patients. The pooled observed effect depicts a Z curve. Two different boundaries of a conventional boundary (*P* < 0.05) and O’Brien–Fleming significance boundary (i.e. trial sequential boundary) were drawn by TSA to determine the preference for antibody or steroid induction group or futility. These two boundaries are symmetrically depicted on both sides of preference. Required information size was calculated to report the sufficient number of participants needed to confirm the preference for the intervention group based on an assumption of 20% relative risk reduction, type I (5%), and type II (20%) errors^17^.

## Results

A total of 2221 publications were identified according to our search strategy. After screening 2221 titles and abstracts, 681 duplicate studies and 1470 irrelevant studies were excluded. Finally, 11 RCTs were included after carefully reviewing the full text. Supplemental Figure S1 shows details of the screening and exclusion process. Of the eleven studies, one trial was published only as a conference abstract^18^, and the outcomes of two trials were pooled in a single report^19^.

The summarized characteristics of included studies are shown in Table 1. Included trials were published between 2001 and 2021. A total of 1683 patients were enrolled with 879 in the antibody induction group and 804 in the steroid induction group. Interleukin-2 receptor antagonists were studied in ten trials^4,5,18–25^: four trials studied basiliximab^4,18,21,23^, and six trials studied daclizumab^5,19,20,22,24,26^. Polyclonal antibodies were studied in one trial which used anti-thymocyte globulin^25^. The distribution of study outcomes across the included trials is shown in Supplemental Table S1.

**Table 1:** Characteristic of the included trials.

Author (Year)	Inclusion criteria	Intervention group drug and regimen	Steroid group drug and regimen	Concomitant drugs
Boillot 2005^5^	Adult primary cadaver LT recipients	Daclizumab 2 mg/kg before reperfusion and 1 mg/kg between day 7–10 Intraoperative steroid bolus 500 mg	Oral prednisolone tapered from 15–20 mg/day on month 1 to 5–10 mg/day on month 3	Tacrolimus
De Simone 2007^18^	HCV-positive adult primary cadaver LT recipients	Basiliximab 20 mg each on day 1 & 4	Corticosteroids for 3 months	Cyclosporine, MMF
Eason 2003^25^	Adult cadaver LT recipients	RATG 1.5 mg/kg before reperfusion and 1.5 mg/kg on posttransplant day 1	Prednisolone 20 mg by day 6 tapered completely by month 3	Tacrolimus, MMF
Kathirvel 2021^4^	Adult primary live donor LT recipients	Basiliximab 20 mg each on day 0 & 4 Intraoperative steroid bolus 500 mg-1 g	Methylprednisolone tapered to 30 mg/day by day 5, tapered completely by month 3	Tacrolimus, Azathioprine
Kato 2001^26^	HCV-positive adult primary LT recipients	Daclizumab 2 mg/kg on day 0, 5 and 1 mg/kg every 2 weeks starting on day 7	Methylprednisolone tapered to 20 mg/day by day 6, tapered completely by month 3	Tacrolimus
Kato 2007^19^	HCV-positive adult primary LT recipients	Daclizumab 2 mg/kg on day 0, 5 and 1 mg/kg every 2 wks starting on day 7	Methylprednisolone tapered to 20 mg/day by day 6, tapered completely by month 3	Tacrolimus, MMF
Klintmalm 2011^24^	HCV-positive adult primary LT recipients	Daclizumab 2 mg/kg on day 0 & 3, 1 mg/kg on day 8 Intraoperative steroid bolus 500 mg	Oral steroid tapered to 5 mg/day by day 90	Tacrolimus, MMF
Lupo 2008^23^	Adult primary cadaver LT recipients	Basiliximab 20 mg on day 0 & 4	Oral prednisolone tapered to 20 mg/day, then tapered completely by day 90	Tacrolimus
Neumann 2012^22^	HCV-positive adult primary cadaver LT recipients	Daclizumab 2 mg/kg on day 0 & between days 7–10	Steroid tapered to 5–10 mg/day by month 3, tapered completely by month 4	Tacrolimus
Spada 2006^21^	Pediatric primary cadaver LT recipients	Basiliximab 10 mg each on day 0 & 4, optional 10 mg dose on day 8–10 if fluid loss from abdominal drains exceeds 70 mL/kg Intraoperative steroid bolus 10 mg/kg	Corticosteroid 2 mg/kg/day until day 6 then tapered completely by month 3–6	Tacrolimus
Washburn 2001^20^	Adult primary LT recipients	Daclizumab 2 mg/kg on day 0 & 14 Intraoperative steroid bolus 1000 mg	Oral corticosteroids tapered to 5 mg/day by week 3–4, tapered completely at 1 year over 3 months	Tacrolimus, MMF

LT = liver transplant, HCV = hepatitis C virus, MMF = mycophenolate mofetil, ATG = antithymocyte globulin.

The risk of bias assessment is shown in Supplemental Figure S2. All studies were at a high risk of bias, mainly due to inadequate blinding of personnel, participants, or the outcome assessor.

**Figure 1:**
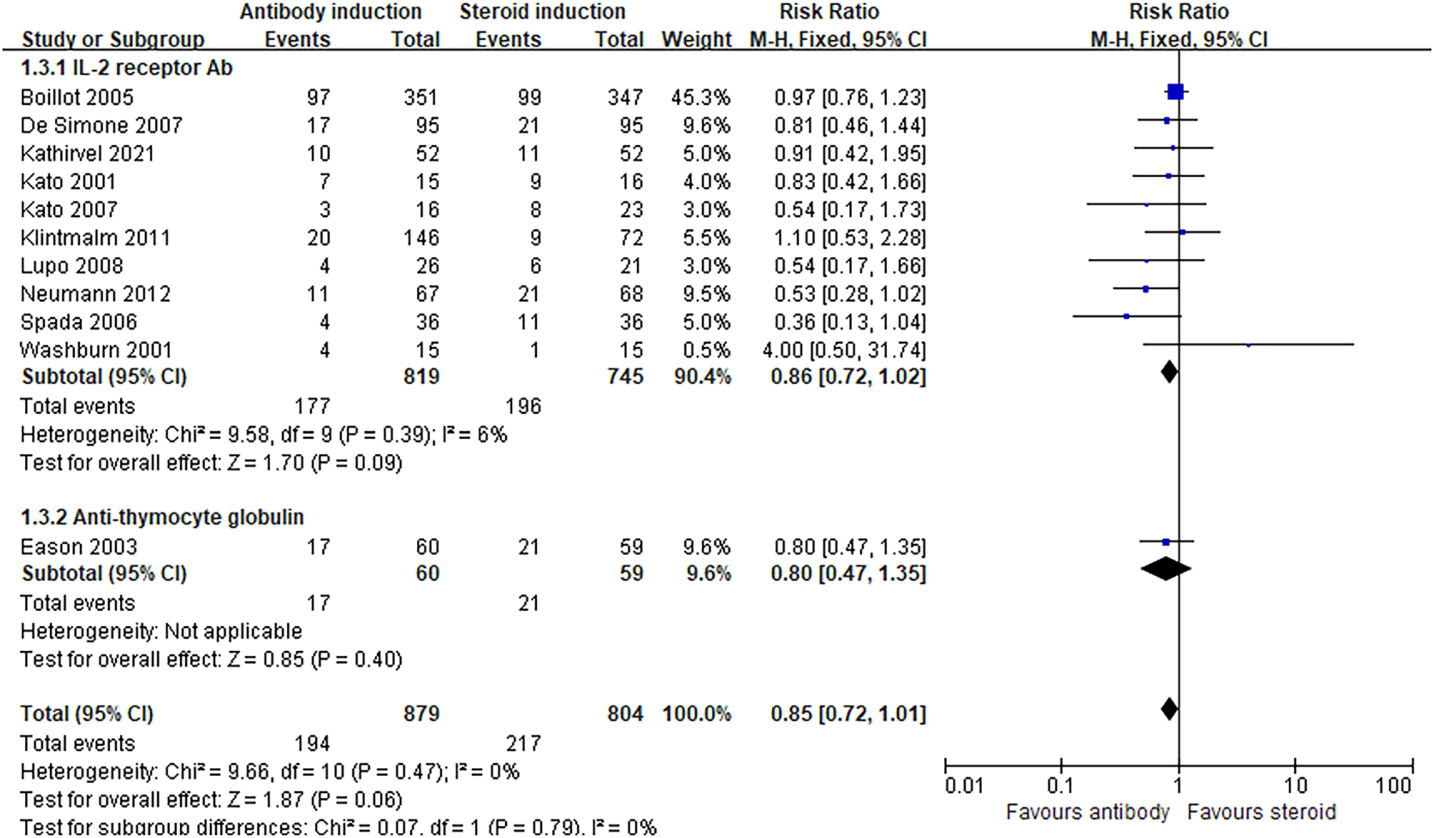
Forest plot of comparison between antibody induction versus corticosteroid induction: Acute rejection.

**Figure 2:**
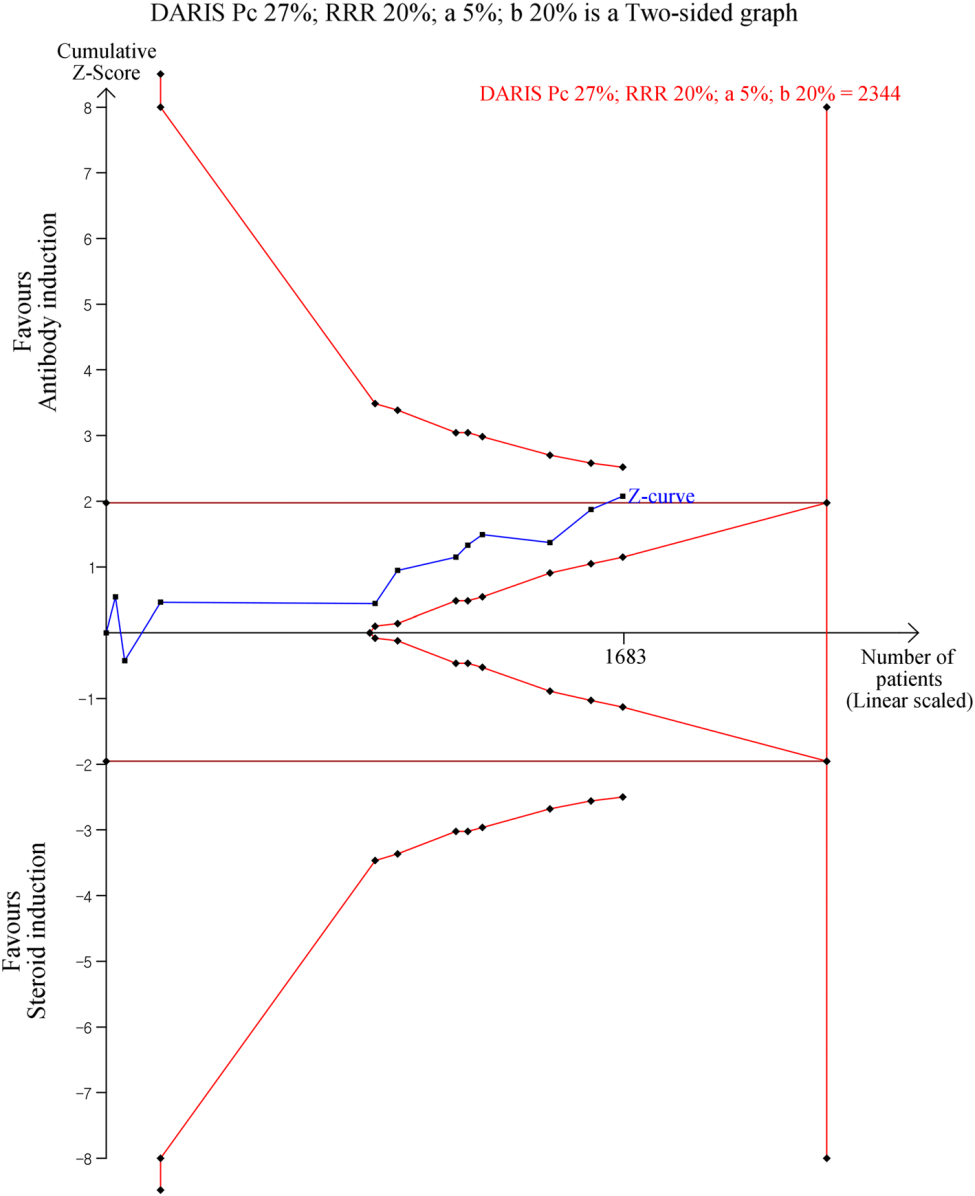
Trial sequential analysis for acute rejection. DARIS = diversity-adjusted required information size, Pc = Probability in the control group, RRR = relative risk reduction, a = alpha error, b = beta-error.

Our primary outcome of the incidence of acute rejection was not significantly different between the antibody and steroid induction groups (MD 0.85, 95% CI 0.72, 1.01, *P* = 0.06: Fig. 1), with no heterogeneity (I^2^ = 0%, *P* = 0.47). TSA demonstrated the cumulative z-curve of the incidence of acute rejection crossed the conventional boundary but did not cross the O’Brien-Fleming significance boundary (Fig. 2). The required information size of 2344 patients was not reached (Fig. 2). Funnel plots of the primary outcome of acute rejection illustrate symmetric properties, indicating the lack of publication bias (Supplemental Figure S3). Egger’s test (*p* = 0.938) also revealed the lack of publication bias.

The results of our secondary outcomes are summarized in Table 2. Of the patient safety-related outcomes, T-cell antibody induction was not associated with an increased risk of all-cause mortality, graft loss including patient death, acute rejection requiring treatment, corticosteroid-resistant rejection, infection, malignancy, and post-transplant lymphoproliferative disorder. There was no significant difference in the total length of hospital stay between groups. TSA of mortality, graft loss, acute rejection requiring treatment, and infection showed that the cumulative z-curve did not cross the O’Brien-Fleming significance boundary and the required information size was not reached (Supplemental Figures S4-S7). T cell antibody induction significantly decreased the risk of CMV infection (RR 0.48, 95% CI 0.33, 0.70, *P* = 0.0002, I^2^ = 3%: Table 2) and HCV recurrence (RR 0.89, 95% CI 0.80, 0.99, *P* = 0.03, I^2^ = 0%: Table 2). TSA for HCV recurrence showed that the cumulative z-curve crossed the O’Brien-Fleming significance boundary, but the required information size of 486 patients was not reached (Supplemental Figure S8).

**Table 2:** Results of the meta-analysis of the secondary outcomes.

Outcomes	Studies	Antibody	Steroid	Effect size [95% CI]*	*P*-value	I^2^
Mortality^4,5,18–25^	10	879	804	0.94 [0.72, 1.24]	0.67	29%
Graft failure^4,5,18–25^	10	879	804	1.07 [0.84, 1.36]	0.58	25%
Acute rejection requiring treatment^4,5,20–24^	7	693	611	0.90 [0.73, 1.11]	0.31	0%
Corticosteroid resistant rejection^4,5,21,22^	4	506	503	0.64 [0.34, 1.18]	0.15	45%
Adverse events^5,19,22,23^	4	475	475	0.97 [0.94, 1.01]	0.13	76%
Infection^4,5,21,23–25^	6	671	587	0.88 [0.72, 1.09]	0.24	0%
CMV infection^5,21,23–25^	5	619	535	0.48 [0.33, 0.70]	0.0002	3%
HCV recurrence^20–25^	6	298	229	0.89 [0.80, 0.99]	0.03	0%
Malignancy^5,20–24^	6	590	582	0.80 [0.33, 1.98]	0.63	0%
PTLD^5,20–23^	5	485	487	1.00 [0.07, 15.38]	1.00	N/A
Total length of hospital stay (days)^4,23^	2	78	73	0.19 [− 3.26, 3.64]	0.91	0%
Renal failure requiring dialysis^4,5,21^	3	439	435	1.29 [0.57, 2.90]	0.54	0%
GFR^4,21,22^	3	118	144	4.77 [1.24, 8.29]	0.008	3%
Serum creatinine level (mmol/L)^5,22,24^	3	508	466	13.93 [7.83, 20.02]	< 0.0001	0%
Diabetes mellitus^4,5,18–25^	10	857	775	0.41 [0.32, 0.54]	< 0.0001	0%
Hyperlipidemia^4,5,18,24^	5	644	566	0.92 [0.71, 1.20]	0.54	70%
Serum cholesterol level (mg/dL)^4,5,20^	4	418	414	− 22.46 [− 28.94, − 15.98]	< 0.0001	22%
Hypertension^4,5,18–21^	6	566	563	0.71 [0.55, 0.90]	0.005	35%

*The data are presented as mean difference or risk ratio with its 95% confidence interval (CI).

CMV = cytomegalovirus; HCV = hepatitis C virus; PTLD = post-transplant lymphoproliferative disorder; GFR = glomerular filtration rate; N/A = not available.

Regarding the renal functional outcomes, no significant difference in renal failure requiring dialysis was found between groups while pooled analysis results for glomerular filtration rate favored T-cell specific antibody induction (MD 4.77, 95% CI 1.24, 8.29, *P* = 0.008, I^2^ = 3%: Table 2) and serum creatinine levels favored corticosteroid induction (MD 13.93, 95% CI 7.83, 20.02, *P* = 0 < 0.00001, I^2^ = 0%: Table 2).

**Figure 3:**
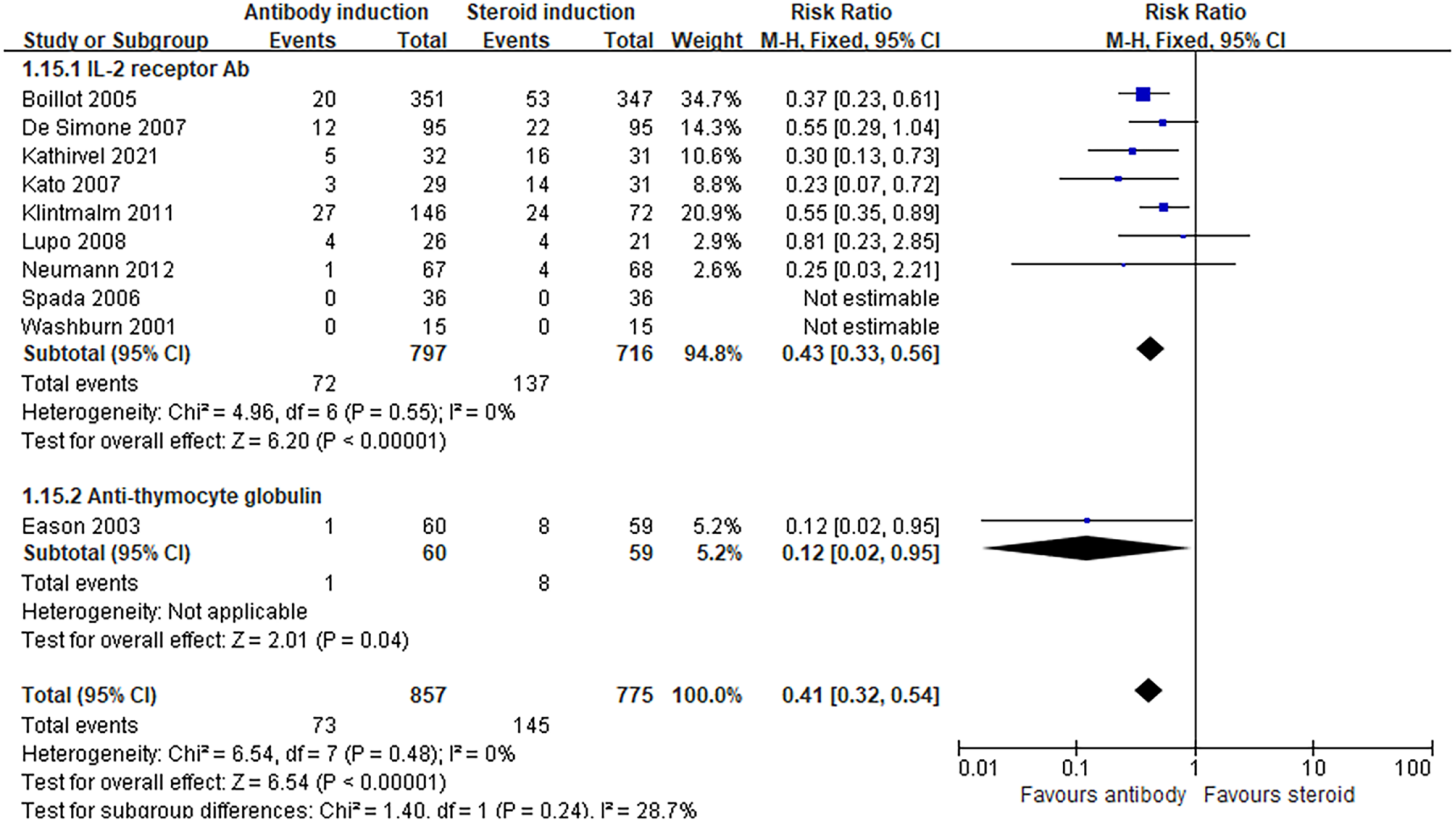
Forest plot of comparison between antibody induction versus corticosteroid induction: new onset Diabetes mellitus.

**Figure 4:**
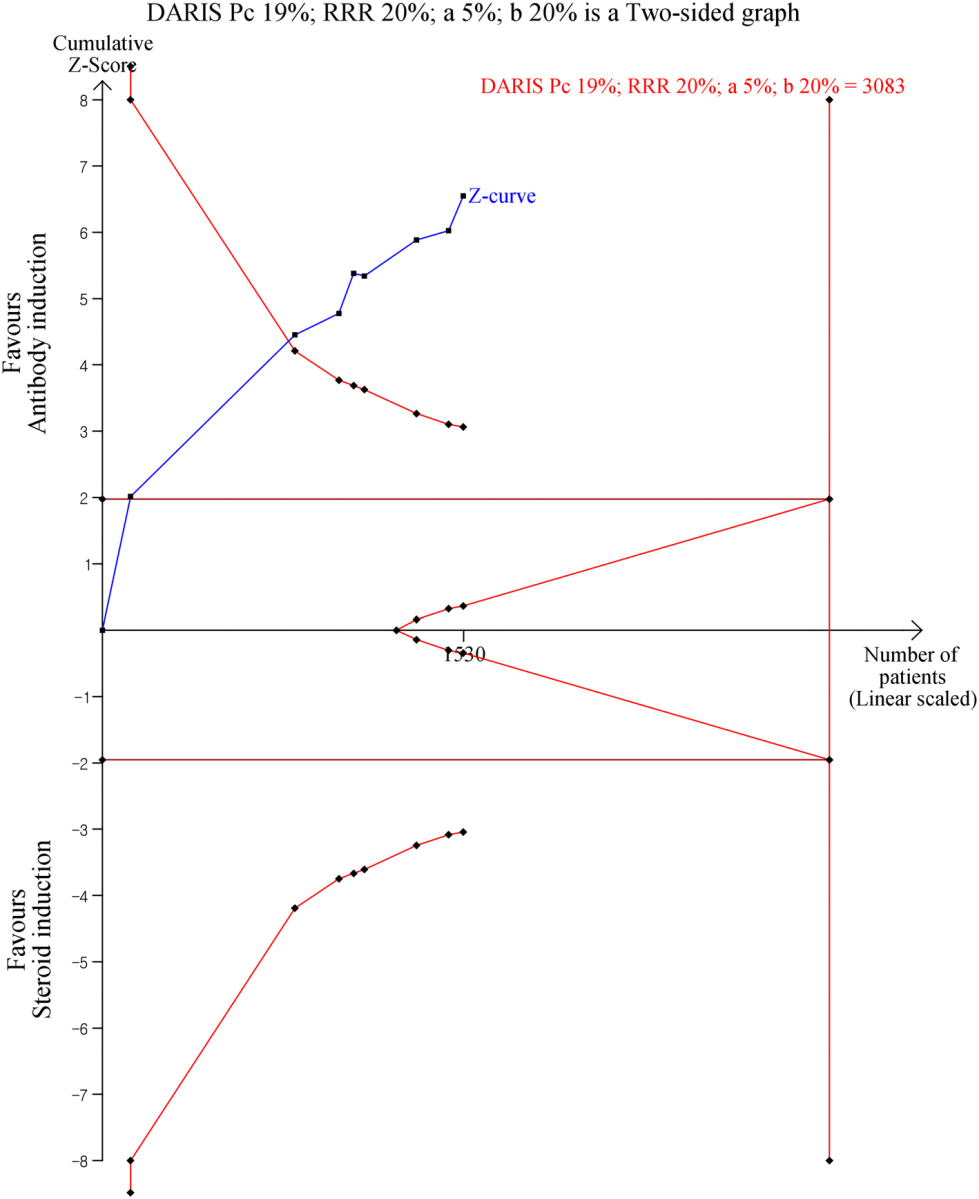
Trial sequential analysis for new-onset diabetes mellitus. DARIS = diversity-adjusted required information size, Pc = Probability in the control group, RRR = relative risk reduction, a = alpha error, b = beta-error.

T-cell antibody induction significantly decreased the risk of metabolic disorders compared to steroids. The risk of diabetes mellitus (RR 0.41, 95% CI 0.32, 0.54, *P* < 0.00001, I^2^ = 0%: Fig. 3), hypertension (RR 0.71, 95% CI 0.55, 0.90, *P* = 0.005, I^2^ = 35%: Table 2), and serum cholesterol levels (MD − 22.46, 95% CI − 28.94, − 15.98, *P* < 0.00001, I^2^ = 22%: Table 2) was significantly lower in the antibody induction group while the risk of hyperlipidemia was similar between groups. TSA for diabetes mellitus showed that the cumulative z-curve crossed both the O’Brien-Fleming significance boundary but the required information size was not reached (Fig. 4). TSA for hypertension showed that trial sequential boundaries were not crossed by the cumulative Z-curve. The calculated required sample size was not reached (Supplemental Figure S9).

Subgroup analysis on different classes of antibodies revealed no significant differences between the interleukin-2 receptor antibody and anti-thymocyte globulin in all outcomes. Subgroup analysis on different formulations of the same class of antibody revealed differences in some outcomes between basiliximab and daclizumab. CMV infection rate (Basiliximab RR 0.83, 95% CI 0.36, 1.91, *P* = 0.66, I^2^ = 0%; Daclizumab RR 0.48, 95% CI 0.30, 0.77, *P* = 0.002, I^2^ = 0%; interaction *P* = 0.26: Table 3) and serum cholesterol levels (Basiliximab MD − 6.25, 95% CI − 13.35, 0.86, *P* = 0.08, I^2^ = 0%; Daclizumab MD − 24.14, 95% CI − 31.13, − 17.14, *P* < 0.001, I^2^ = 2%; interaction *P* = 0.0004: Table 3) were significantly reduced only in the daclizumab group. Hypertension (Basiliximab RR 0.54, 95% CI 0.37, 0.78, *P* = 0.001, I^2^ = 48%; Daclizumab RR 0.86, 95% CI 0.62, 1.18, *P* = 0.34, I^2^ = 0%; interaction *P* = 0.07: Table 3) and hyperlipidemia (Basiliximab RR 0.47, 95% CI 0.27, 0.79, *P* = 0.005, I^2^ = 0%; Daclizumab RR 1.25, 95% CI 0.92, 1.72, *P* = 0.16, I^2^ = 0%; interaction *P* = 0.002: Table 3) were only significantly reduced in the basiliximab group. Subgroup analysis according to the administration of intraoperative steroid bolus revealed differences in some outcomes between the groups with and without intraoperative bolus. Acute rejection, adverse events, and hypertension were significantly reduced only in the no-bolus group, while GFR and serum creatinine were significantly increased only in the bolus group.

**Table 3:** Results of subgroup analyses.

Outcomes	Interleukin-2 receptor antibody versus steroid					Anti-thymocyte globulin versus steroid					*P*-value†
	Studies	Antibody	Steroid	Effect size [95% CI]*	*P*-value	Studies	Antibody	Steroid	Effect size [95% CI]*	*P*-value	
1. Subgroup analysis classified by type of T-cell specific antibody											
Mortality^4,5,18–25^	9	819	745	0.93 [0.69, 1.24]	0.60	1	60	59	1.08 [0.69, 1.24]	0.84	0.71
Graft failure^4,5,18–25^	9	819	745	1.09 [0.85, 1.41]	0.50	1	60	59	0.90 [0.43, 1.88]	0.78	0.63
Acute rejection^4,5,18–26^	10	819	745	0.86 [0.72, 1.02]	0.09	1	60	59	0.80 [0.47, 1.35]	0.40	0.79
Infection^4,5,21,23–25^	5	611	528	0.89 [0.72, 1.10]	0.28	1	60	59	0.85 [0.44, 1.63]	0.63	0.90
CMV infection^5,21,23–25^	4	559	476	0.54 [0.36, 0.82]	0.003	1	60	59	0.21 [0.06, 0.70]	0.01	0.14
HCV recurrence^20–25^	5	269	196	0.89 [0.80, 1.00]	0.05	1	60	59	0.85 [0.60, 1.21]	0.38	0.81
Diabetes mellitus^4,5,18–25^	9	797	716	0.43 [0.33, 0.56]	< 0.001	1	60	59	0.12 [0.02, 0.95]	0.04	0.24

Outcomes	Basiliximab versus Steroid					Daclizumab versus Steroid					*P*-value†
	Studies	Antibody	Steroid	Effect size [95% CI]*	*P*-value	Studies	Antibody	Steroid	Effect size [95% CI]*	*P*-value	
2. Subgroup analysis classified by type of interleukin-2 receptor antibody											
Mortality^4,5,19–25^	4	209	204	0.67 [0.39, 1.15]	0.15	5	610	541	1.16 [0.82, 1.65]	0.40	0.09
Graft failure^4,5,19–25^	4	209	204	0.89 [0.57, 1.41]	0.63	5	610	541	1.20 [0.88, 1.63]	0.26	0.30
Acute rejection^4,5,19–25^	4	209	204	0.70 [0.47, 1.03]	0.07	6	610	541	0.91 [0.75, 1.11]	0.36	0.23
Acute rejection requiring treatment^4,5,21–25^	3	114	109	0.64 [0.37, 1.09]	0.10	4	453	439	0.95 [0.77, 1.18]	0.63	0.18
Corticosteroid resistant rejection^4,5,22,23^	2	88	88	2.00 [0.19, 21.38]	0.57	2	418	415	0.58 [0.30, 1.10]	0.10	0.32
Infection^4,5,22,24,25^	3	114	109	0.75 [0.54, 1.04]	0.08	2	497	419	0.98 [0.74, 1.30]	0.88	0.23
CMV infection^5,22,24,25^	2	62	57	0.83 [0.36, 1.91]	0.66	2	497	419	0.30 [0.36, 0.77]	0.002	0.26
HCV recurrence^21–25^	2	48	44	0.77 [0.54, 1.12]	0.17	3	221	152	0.90 [0.80, 1.02]	0.09	0.43
Renal failure requiring dialysis^4,5,22^	2	88	88	3.00 [0.33, 27.50]	0.33	1	351	347	1.10 [0.45, 2.67]	0.84	0.41
GFR^4,22,23^	2	88	88	5.38 [1.71, 9.04]	0.004	1	30	56	− 2.50 [− 15.14, 10.14]	0.70	0.24
Diabetes mellitus^4,5,19–25^	4	189	183	0.48 [0.30, 0.77]	0.002	5	608	533	0.41 [0.29, 0.56]	< 0.001	0.57
Hyperlipidemia^4,5,19,22,25^	3	183	183	0.47 [0.27, 0.79]	0.005	2	497	419	1.25 [0.92, 1.72]	0.16	0.002
Serum cholesterol level (mg/dL)^4,5,21,22^	2	88	88	− 6.25 [− 13.35, 0.86]	0.08	2	366	362	− 24.14 [− 31.13, − 17.14]	0.0004	< 0.001
Hypertension^4,5,19–22^	3	173	167	0.54 [0.37, 0.78]	0.001	3	393	396	0.86 [0.62, 1.18]	0.34	0.07

Outcomes	No intraoperative steroid bolus					Intraoperative steroid bolus					*P*-value†
	Studies	Antibody	Steroid	Effect size [95% CI]*	*P*-value	Studies	Antibody	Steroid	Effect size [95% CI]*	*P*-value	
3. Subgroup analysis classified by administration of intraoperative steroid bolus											
Mortality^4,5,18–25^	5	279	282	1.12 [0.75, 1.68]	0.58	5	600	522	0.82 [0.57, 1.19]	0.30	0.27
Graft loss^4,5,18–25^	5	279	282	1.19 [0.83, 1.70]	0.34	5	600	522	0.99 [0.71, 1.37]	0.58	0.45
Acute rejection^4,5,18–25^	6	279	282	0.70 [0.53, 0.93]	0.01	5	600	522	0.95 [0.77, 1.17]	0.62	0.09
Acute rejection requiring treatment^4,5,20–24^	2	93	89	0.73 [0.35, 1.52]	0.41	5	600	522	0.91 [0.73, 1.14]	0.43	0.57
Corticosteroid resistant rejection^4,5,21,22^	1	67	68	2.03 [0.38, 10.71]	0.40	3	439	435	0.52 [0.26, 1.02]	0.06	0.14
Adverse events^5,19,22,23^	3	124	128	0.84 [0.74, 0.95]	0.006	1	351	347	1.00 [0.97, 1.04]	0.84	0.006
Infection^4,5,21,23–25^	2	86	80	0.93 [0.53, 1.64]	0.81	4	585	507	0.88 [0.70, 1.09]	0.24	0.84
CMV infection^5,21,23–25^	2	86	80	0.38 [0.16, 0.88]	0.02	3	533	455	0.51 [0.33, 0.70]	0.002	0.53
HCV recurrence^20–25^	3	108	109	0.89 [0.78, 1.02]	0.10	3	190	120	0.88 [0.74, 1.06]	0.17	0.92
Malignancy^5,20–24^	2	93	93	1.52 [0.26, 8.82]	0.64	4	497	493	0.63 [0.21, 1.84]	0.39	0.40
GFR ^4,21,22^	1	30	56	− 2.50 [− 15.14, 10.14]	0.70	2	88	88	5.38 [1.71, 9.04]	0.004	0.24
Serum creatinine level (mg/dL)^5,22,24^	1	30	56	17.60 [− 11.57, 46.77]	0.24	2	478	410	13.76 [7.53, 19.99]	< 0.001	0.80
Diabetes mellitus^4,5,18–25^	5	277	274	0.40 [0.25, 0.64]	< 0.001	5	580	501	0.42 [0.30, 0.58]	< 0.001	0.86
Hyperlipidemia^4,5,18,21,24^	1	95	95	0.25 [0.03, 2.20]	0.21	3	549	471	0.96 [0.73, 1.25]	0.77	0.23
Hypertension^4,5,18–21^	2	122	129	0.69 [0.49, 0.98]	0.04	4	444	434	0.72 [0.55, 1.01]	0.06	0.89

*The data are presented as mean difference or risk ratio with its 95% confidence interval (CI).

^†^*P*-value for interaction.

CMV, cytomegalovirus; HCV , hepatitis C virus; GFR, glomerular filtration rate.

Meta-regression analyses showed that the differences in recipient age, sex ratio, laboratory MELD score, and cold ischemia time between the antibody induction group and steroid induction group did not significantly influence acute rejection (Supplemental Figure S10).

Results from sensitivity analyses comparing a random-effects model to a fixed-effects model showed no difference for most outcomes. However, while the risk of HCV recurrence was significantly lower in the antibody induction group when using a fixed-effects model (RR 0.89, 95% CI 0.80, 0.99, I^2^ = 0%, *P*-value = 0.03: Table 2), this was not the case when using the random-effects model (RR 0.90, 95% CI 0.81, 1.00, I^2^ = 0%, *P*-value = 0.05: Supplemental Table S2).

The quality of evidence evaluated by the GRADE system was summarized for all our study outcomes in Supplemental Table S3. Most of our outcomes were at moderate to very low quality of evidence.

## Discussions

In this meta-analysis, we sought to compare the efficacy and safety of T-cell specific antibody induction and corticosteroid induction in liver transplant recipients. Our pooled analysis showed that T-cell specific antibody induction did not decrease the incidence of acute rejection compared to corticosteroid induction. However, antibody induction significantly reduced CMV infection, HCV recurrence, DM, and hypertension while the incidence of graft loss and mortality was similar. There was no significant difference in the incidence of renal failure requiring dialysis, but antibody induction significantly decreased serum cholesterol levels. Although the required information size was not reached for all outcomes as revealed by TSA, cumulative evidence crossed the trial sequential boundary for DM and HCV recurrence. However, our results should be interpreted cautiously given the low quality of evidence for most outcomes, high risk of bias and significant heterogeneity of study protocols of the included RCTs, and insufficient information size.

No significant difference in the biopsy-proven acute rejection was found between the antibody induction and corticosteroid induction. We obtained the same results when comparing the subgroups of antibodies with corticosteroids; interleukin-2 receptor antibodies, polyclonal antibodies, basiliximab, and daclizumab These results were consistent in a recent meta-analysis^10^. Therefore, we could conclude that the use of antibody induction was not associated with an increased risk of acute rejection, which may allow the substitution of corticosteroid induction with antibody induction.

Our enrolled population had heterogeneity regarding the underlying liver disease. It is well-known that the risk of acute rejection varies depending on the indication of liver transplantation, especially autoimmune liver disease^27^. In patients with autoimmune hepatitis, acute cellular rejection (ACR) or steroid-resistant ACR may occur more frequently than those with other liver diseases^28,29^. Therefore, it is possible that the effect of T-cell specific antibody induction with steroid avoidance or minimization on the incidence of acute rejection may differ in the subgroups with autoimmune hepatitis. It is important to consider the underlying liver disease when interpreting the results of our meta-analysis, and further studies are needed to evaluate the effect of T-cell specific antibody induction with steroid avoidance or minimization on the incidence of acute rejection across different subgroups of liver transplantation.

Patients undergoing liver transplantation with autoimmune diseases such as autoimmune hepatitis, biliary cirrhosis, and primary sclerosing cholangitis, there is debate regarding the benefit or concerns of steroid avoidance or minimization in the immunosuppression regimen^30,31^. Patients with autoimmune hepatitis receive corticosteroids prior to transplant. The issue is whether weaning steroids after transplantation contributes to the risk of recurrent autoimmune hepatitis. A previous study of 74 patients with autoimmune liver disease reported acceptable rates of survival and acute cellular rejection without corticosteroid maintenance dose^32^. Another study of 66 patients showed that the association between steroid withdrawal and recurrence of autoimmune hepatitis was not significant^33^. However, the first reported cases of autoimmune hepatitis occurred during weaning or withdrawal of steroid or on a background of long-term calcineurin inhibitor monotherapy^31^. Furthermore, previous studies of steroid withdrawal did not include a separate analysis for patients with autoimmune diseases or excluded such patients because of concerns regarding acute rejection. The use of steroid avoidance or minimization in patients with autoimmune diseases should be evaluated on a case-by-case basis, given the risk of acute rejection and complications associated with steroid-free regimens. We could not provide subgroup analysis for these patients because our meta-analysis also included only a few trials with a small proportion of patients with autoimmune liver disease^21,25^.

Meanwhile, we found some significant benefits of antibody induction. The risk of DM was significantly decreased in the T-cell antibody induction group, which was consistent in previous meta-analyses^8,10,11^ and our subgroup analyses of different immunosuppressive regimens with corticosteroids. Although the required information size was not reached, the cumulative Z-curve crossed the O’Brien-Fleming boundary for benefit.

The risk of CMV infection was also significantly decreased in antibody induction which was consistent in a recent meta-analysis^10^, while not in another previous meta-analysis^8^. This result was consistent in most of our subgroup analysis but there was no difference in the risk of CMV infection when basiliximab was compared to corticosteroids. For this subgroup of basiliximab, the pooled estimate was imprecise and included only two studies.

Furthermore, the risk of HCV recurrence was significantly decreased in the T-cell antibody induction group. Although the required information size was not reached in TSA, the cumulative estimate crossed the trial sequential boundary for benefit. However, none of our subgroup analyses showed significant results for this outcome, which means further studies are required to interpret significant results for HCV recurrence at the subgroup levels. It should be noted that the current use of antivirals both pre and post-transplant may limit the relevance of our findings in the context of HCV recurrence^34–36^. The American Society of Transplantation Consensus Conference on the use of hepatitis C viremic donors in solid organ transplantation has provided guidelines on the use of HCV-positive organs for transplantation^37^. Although 16.9% of HCV-infected liver recipients receive liver graft from HCV-positive donors, the possible positive impact of steroid avoidance in hepatitis C viremic donors has not been studied. Furthermore, a previous meta-analysis reported different results regarding HCV recurrence^10^, which may be because our updated meta-analysis added a recent trial. The risk of hypertension was significantly decreased in the T-cell antibody induction group. However, some of our subgroup analyses did not show consistent results when comparing daclizumab to corticosteroids and when comparing antibody induction with an intraoperative steroid bolus to corticosteroids.

In patients undergoing liver transplantation with autoimmune diseases such as autoimmune hepatitis, biliary cirrhosis, and primary sclerosing cholangitis, there is debate regarding the benefit or concerns of steroid avoidance or minimization in the immunosuppression regimen^30,31^. Patients with autoimmune hepatitis receive corticosteroids prior to transplant. The issue is whether weaning steroids after transplantation contributes to the risk of recurrent autoimmune hepatitis. A previous study of 74 patients with autoimmune liver disease reported acceptable rates of survival and acute cellular rejection without corticosteroid maintenance dose^32^. Another study of 66 patients showed that the association between steroid withdrawal and recurrence of autoimmune hepatitis was not significant^33^. However, the first reported cases of autoimmune hepatitis occurred during weaning or withdrawal of steroid or on a background of long-term calcineurin inhibitor monotherapy^31^. Furthermore, previous studies of steroid withdrawal did not include a separate analysis for patients with autoimmune diseases or excluded such patients because of concerns regarding acute rejection. The use of steroid avoidance or minimization in patients with autoimmune diseases should be evaluated on a case-by-case basis, given the risk of acute rejection and complications associated with steroid-free regimens. We could not provide subgroup analysis for these patients because our meta-analysis also included only a few trials with a small proportion of patients with autoimmune liver disease^21,25^.

Previous meta-analyses reported no significant differences in renal function between antibody induction and corticosteroids^8,10,11^. This was consistent in our meta-analysis regarding renal failure requiring dialysis. However, in the T-cell specific antibody group, GFR and serum creatinine levels were significantly increased. These results are conflicting with each other because antibody induction was favorable regarding an increase in GFR and steroid induction was favorable regarding the outcome of serum creatinine. Postoperative use of calcineurin inhibitor and its different trough level may act as a confounding factor and we cannot conclusively say the pure effect of immunosuppression regimen on renal function. Also, given the effect size for these outcomes does not correspond to the levels of clinical significance, the high risk of publication bias due to the small number of studies—three for each outcome—included in the analysis, and the insignificance of the most direct outcome of renal failure requiring dialysis, we believe that renal function may not be significantly different between groups.

Our meta-analysis results suggest that T-cell specific antibody induction with steroid avoidance or minimization could lead to a significant reduction in metabolic complications, including diabetes mellitus and hypertension when compared to corticosteroid induction. NASH patients had a higher risk of developing metabolic syndrome after liver transplantation, which can lead to long-term complications such as cardiovascular disease^38^. Therefore, there is a potential relevance of the metabolic advantage of steroid avoidance to individuals with NASH. However, we could not find any previous study that compared the metabolic outcomes between steroid-free immunosuppression and steroid-based immunosuppression in NASH patients undergoing liver transplantation. Among our included trials, only one trial enrolled a small number of patients with NASH^4^, we could not perform subgroup analysis due to mixed baseline liver diseases of that study. Further studies are required to evaluate whether the benefit of metabolic outcomes by steroid-free immunosuppression is greater in patients with NASH compared to those without.

There were no significant differences in other secondary outcomes including mortality, graft loss, acute rejection requiring treatment, corticosteroid-resistant rejection, and infection, which was consistent in the subgroups comparing different antibodies and immunosuppressive regimens with corticosteroids. Other outcomes such as adverse events, malignancy, the total length of hospital stay, and hyperlipidemia were also not significant. Two previous meta-analyses reported the same results^8,10^. While serum cholesterol levels were significantly higher in the corticosteroid group, we deemed this difference insignificant due to the mean cholesterol levels in the corticosteroid group (about 180 mg/dL) being low enough to ignore the difference.

Three previous meta-analyses reported the use of T-cell specific antibody induction for corticosteroid avoidance^8,10,11^. However, only one meta-analysis included RCTs comparing T-cell specific antibody induction with corticosteroids with identical concomitant immunosuppressive regimens between groups^10^. Wang et al.^8^ and Goralczyk et al.^11^ analyzed only studies on interleukin-2 receptor antibodies. Also, Wang et al. compared studies with different immunosuppressive regimens other than antibodies and corticosteroids, making it impossible to compare the pure impact of antibody induction versus corticosteroid induction. Goralczyk et al. included non-randomized studies, decreasing the quality of evidence.

Compared to previous meta-analyses^8,10,11^, our study analyzed additional outcomes such as acute rejection requiring treatment, length of hospital stay, and serum cholesterol levels. We also performed an additional subgroup analysis to address the effect of omitting the intraoperative bolus dose of corticosteroids. Meta-regression analyses of our primary outcome using recipient age, sex ratio, MELD score, and cold ischemia time were also performed. Regarding secondary outcomes, we observed antibody induction reduced the risk of hypertension and serum cholesterol levels. Compared to a previous meta-analysis^10^, the quality of evidence for CMV infection, HCV recurrence, and DM has been increased to ‘moderate’ due to our updated analysis.

Our results should be interpreted cautiously due to the following important limitations.

Firstly, daclizumab has been withdrawn from the market after reports of autoimmune encephalitis in Europe^39^. Analysis including trials with daclizumab now has a limited value.

Secondly, due to the high risk of bias and imprecision, the majority of our outcomes were at a ‘low’ or ‘very low’ quality of evidence. The majority of the included trials had a high risk of bias due to not blinding participants, personnel, and outcome assessors^4,5,18–24^. Thirdly, significant heterogeneity of study protocols should also be considered. Most RCTs differed in inclusion criteria, the type and dose of antibody or corticosteroid, and the type and dose of concomitant other immunosuppressive drugs. Fourthly, the required information size was not reached for all outcomes in TSA and the O’Brien-Fleming boundary for benefit was not crossed for CMV infection and hypertension. Fifthly, we could not find means and standard deviations for recipient age, cold ischemia time, and total hospital stay in one RCT^23^. We used medians as means and assumed standard deviations by dividing ranges by four. This method could be used only when the distribution of our variable follows the normal distribution.

In conclusion, according to our updated meta-analysis, T-cell specific antibody induction appears to significantly reduce cytomegalovirus infection and HCV recurrence and metabolic complications including DM and hypertension when compared to corticosteroid induction. However, most included RCTs were assigned a high risk of bias, and the required sample size was not reached for all outcomes. Furthermore, included RCTs were heterogeneous in the immunosuppressive regimens and study protocols. Therefore, additional RCTs with high quality and adequate study power are still needed to assess the efficacy and safety of T-cell specific antibody induction for a higher quality of evidence.

## Supplementary Information

Supplementary Information 1.

Supplementary Information 2.

## Data Availability

All other data is available in the Supplementary Information files. Any further information is available upon request from the corresponding author.

## Abbreviations

ATG: Antithymocyte globulin
CI: Confidence interval
DM: Diabetes mellitus
GRADE: Grading of recommendations, assessment, development, and evaluation
HCV: Hepatitis C
MD: Mean difference
RR: Risk ratio
TSA: Trial sequential analysis
