# Supplementary material for: T-cell specific antibody induction versus corticosteroid induction immunosuppression for liver transplant recipients: a meta-analysis
Source: Sci Rep. 2023 Apr 28;13:6951. doi: 10.1038/s41598-023-32972-z (PMC10147598; doi:10.1038/s41598-023-32972-z)
Supplement: Supplementary file 1 — Supplementary Information 1. [file 41598_2023_32972_MOESM1_ESM.docx]

**T-cell specific antibody induction versus corticosteroid induction immunosuppression for liver transplant recipients: a meta-analysis**

**: Supplementary information**

**Supplemental Figure S1.** Study flow diagram.

**Supplemental Figure S2.** Risk of bias assessment of the included studies (version 2.0).

**Supplemental Figure S3.** Funnel plot of comparison: Acute rejection.

**Supplemental Figure S4.** Trial sequential analysis for mortality.

**Supplemental Figure S5.** Trial sequential analysis for graft loss.

**Supplemental Figure S6.** Trial sequential analysis for acute rejection requiring treatment.

**Supplemental Figure S7.** Trial sequential analysis for infection.

**Supplemental Figure S8.** Trial sequential analysis for HCV recurrence.

**Supplemental Figure S9.** Trial sequential analysis for hypertension.

**Supplemental Figure S10.** Results of meta-regression analyses.

**Supplemental Table S1.** Distribution of study outcomes across the included trials.

**Supplemental Table S2.** Results of the sensitivity analyses using a random-effects model.

**Supplemental Table S3.** Quality of evidence (GRADE approach).

**Supplemental Figure S1.** PRISMA flow diagram.

**Identification of studies via databases and registers**

Records identified from:

MEDLINE (n = 310)

EMBASE (n = 651)

CENTRAL (n = 1260)

Records removed *before screening*:

Duplicate records removed (n = 681)

**Identification**

Records excluded (n = 1470)

• Animal study (n = 33)

• Case report or case series (n = 212)

• Pediatric study (n = 71)

• Review article (n = 49)

• Meta-analysis (n = 15)

• Study Protocol (n = 33)

• Study of kidney transplantation (n = 84)

• Study of stem cell transplantation (n = 98)

• Study of other subjects (n = 430)

• Observational study (n = 445)

Records screened

(n = 1540)

**Screening**

Reports not retrieved (n = 0)

Reports sought for retrieval

(n = 68)

Reports excluded (n = 58):

• Retrospective/ Non-randomized/ Single-arm study (n=20)

• Different regimen other than steroid and antibody between the groups (n = 6)

• Concomitant steroid use in antibody induction group (n = 13)

• Irrelevant study (n = 17)

• Secondary analysis of included study (n = 1)

Reports assessed for eligibility

(n = 68)

Studies included in review

(n = 11)

Reports of included studies

(n = 11)

**Included**

**Supplemental Figure S2.** Risk of bias assessment of the included studies (version 2.0).


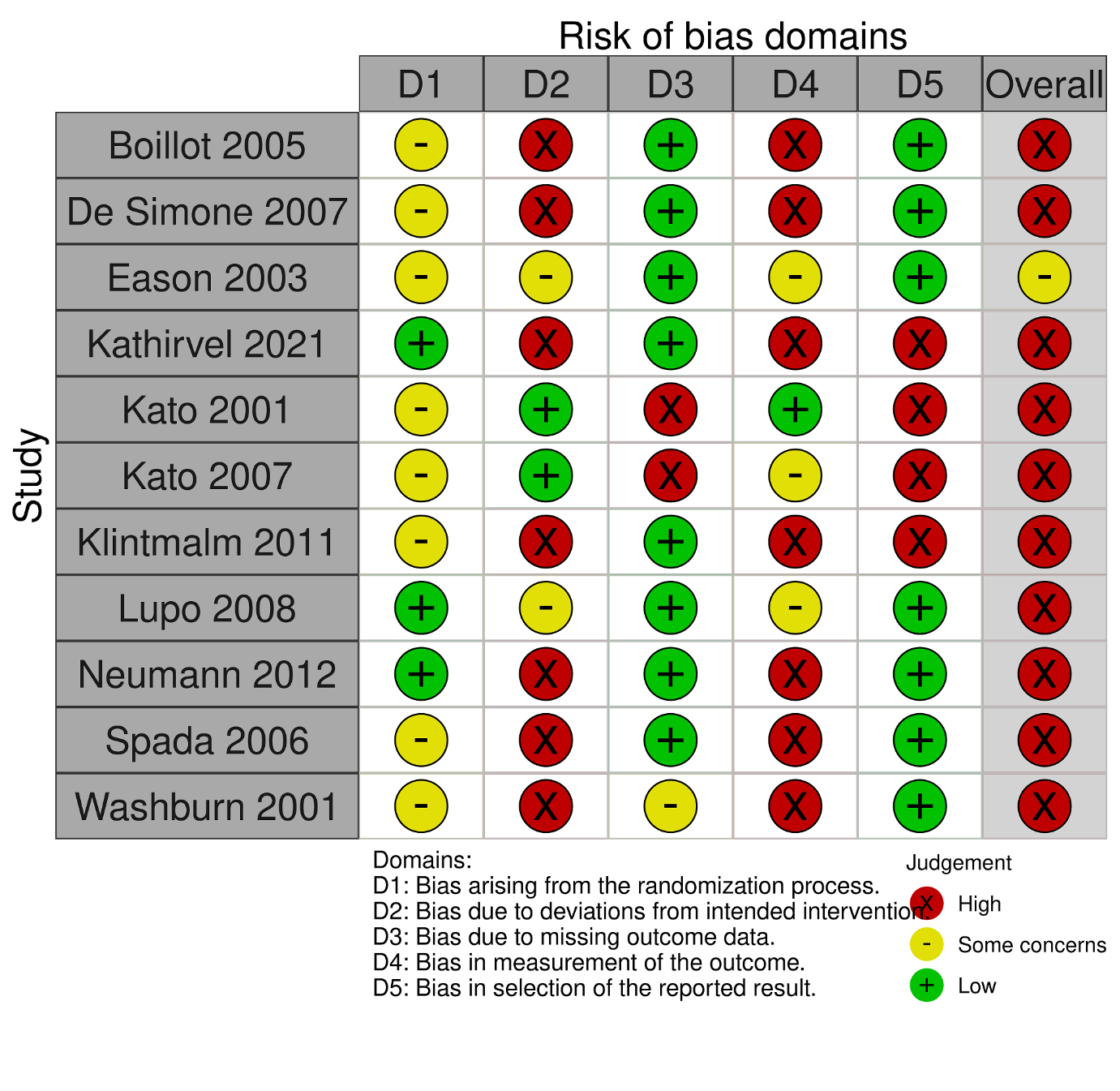


**Supplemental Figure S3.** Funnel plot of comparison: Acute rejection.

On the y-axis standard error of the relative risk of the outcome of interest (measure of trial size) was plotted as a function, on the x-aixs, of the relative risk of the outcome.

**
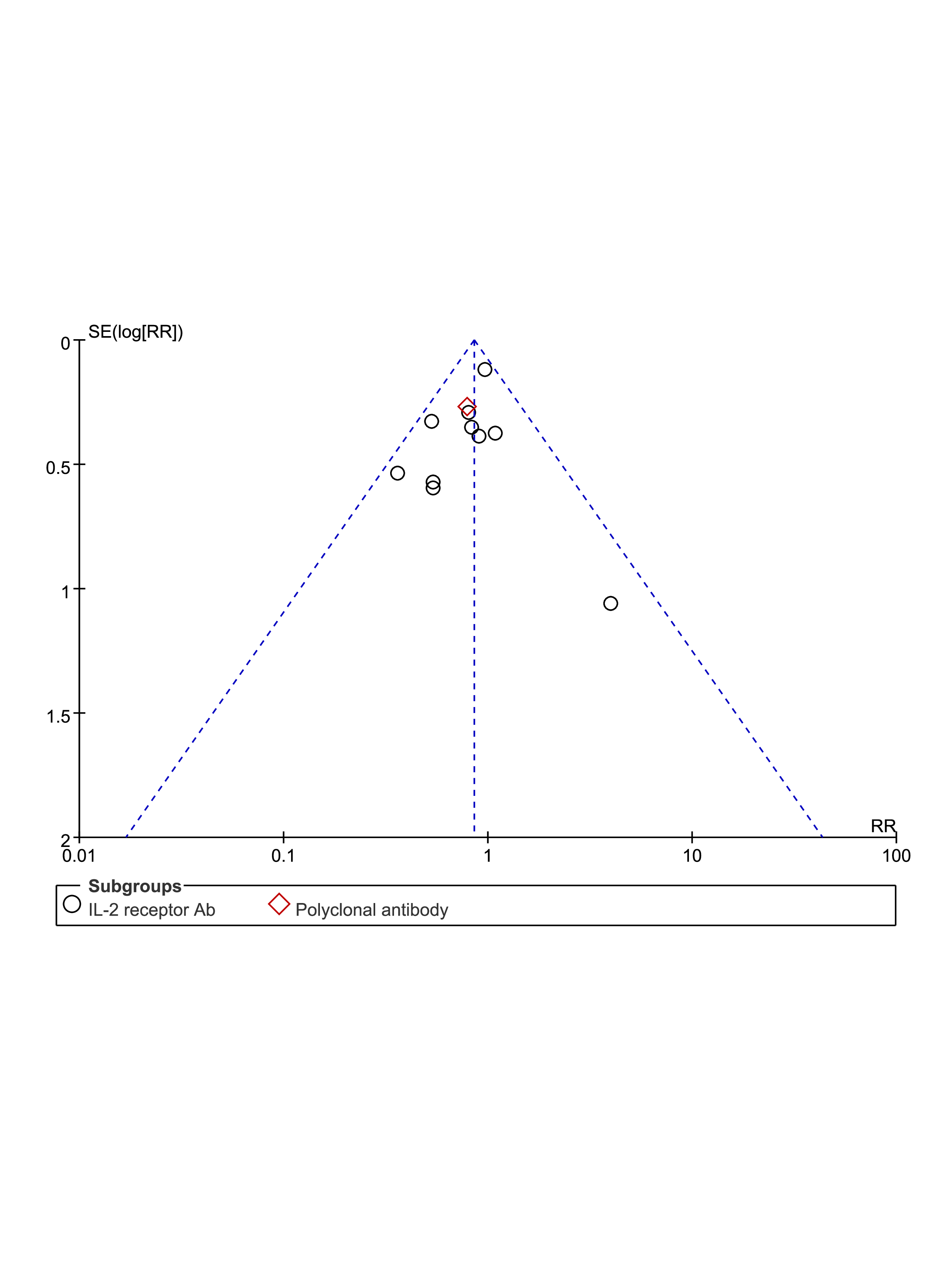
**

**Supplemental Figure S4.** Trial sequential analysis for mortality.


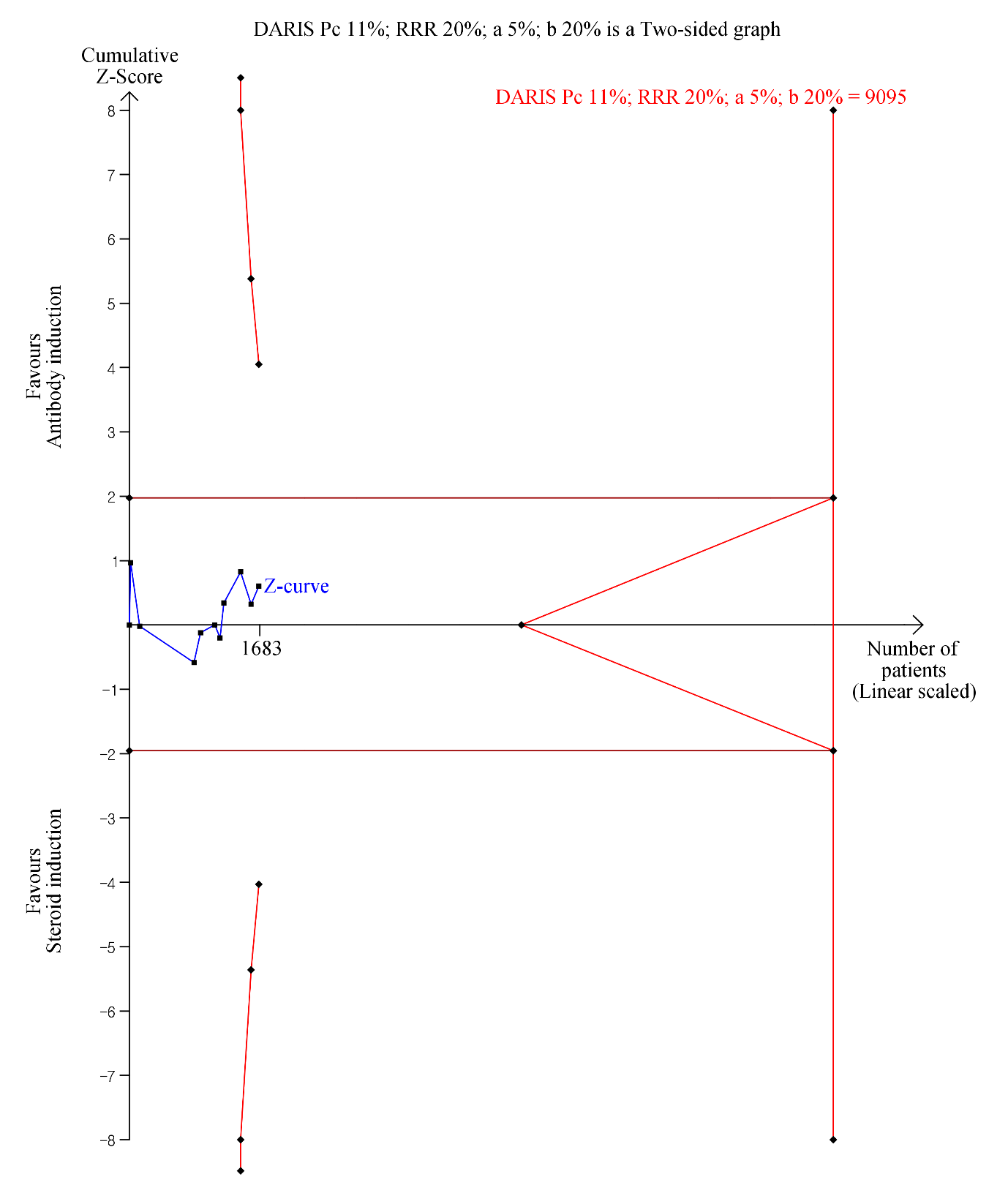


**Supplemental Figure S5.** Trial sequential analysis for graft loss.


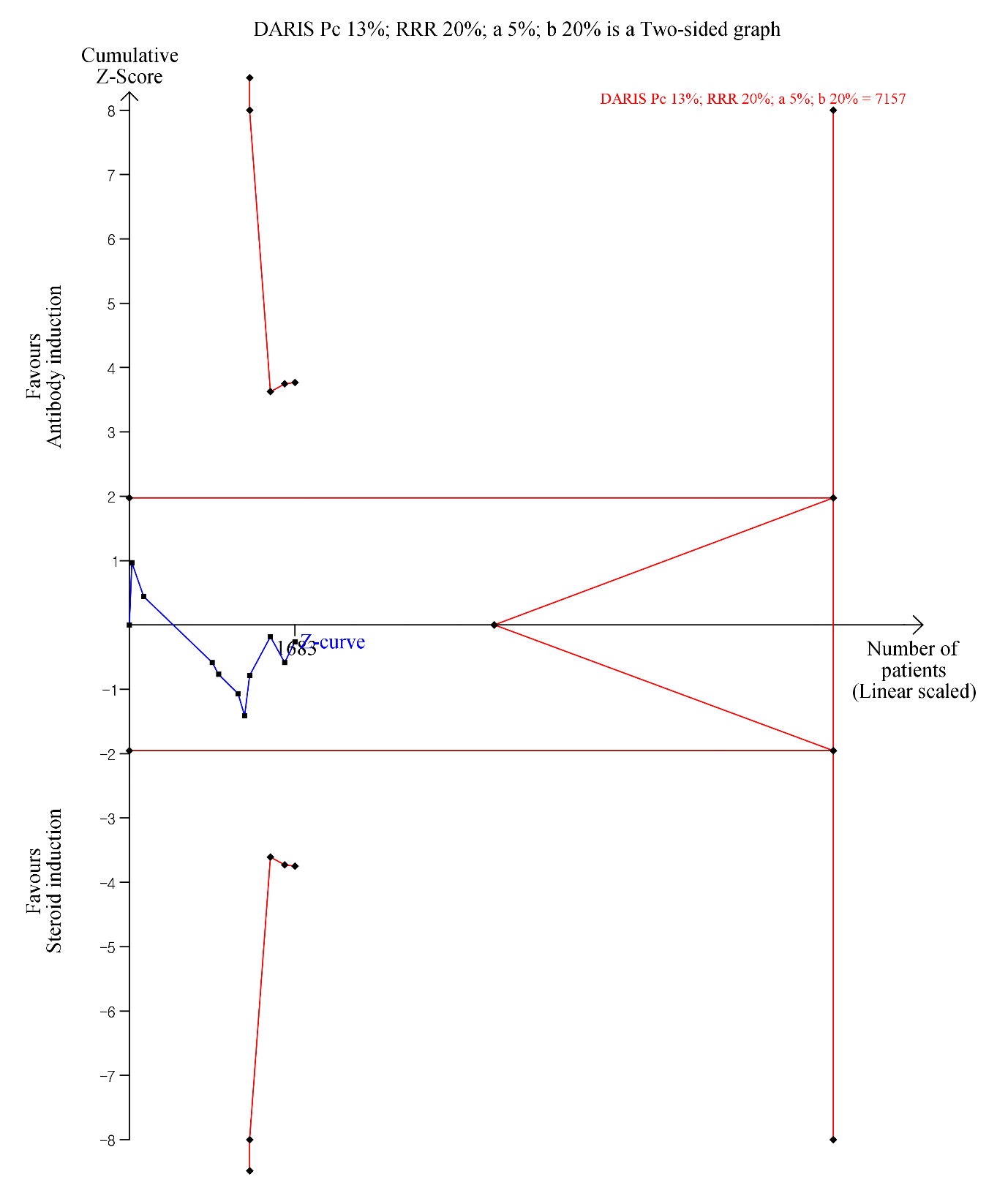


**Supplemental Figure S6.** Trial sequential analysis for acute rejection requiring treatment.


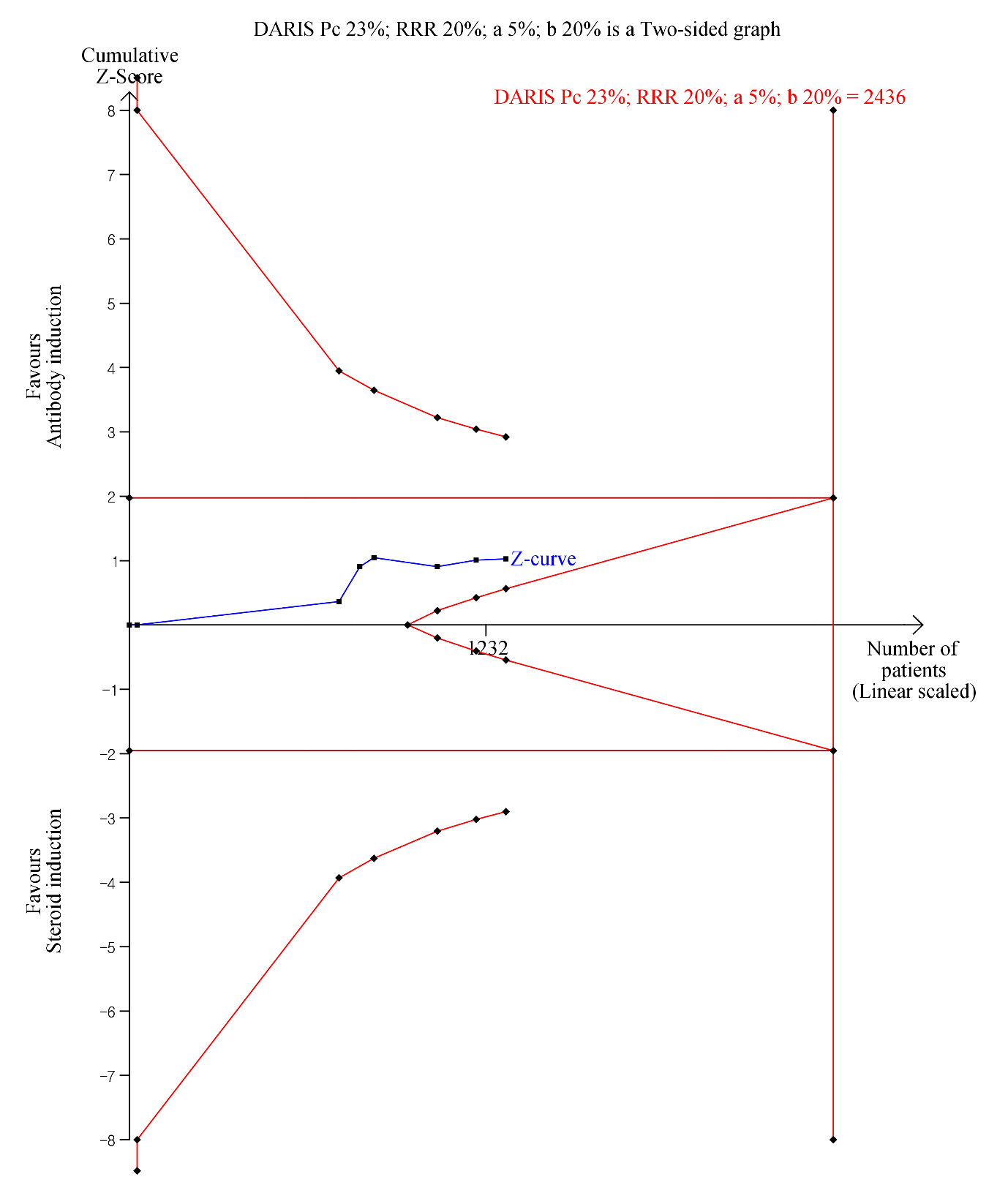


**Supplemental Figure S7.** Trial sequential analysis for infection.


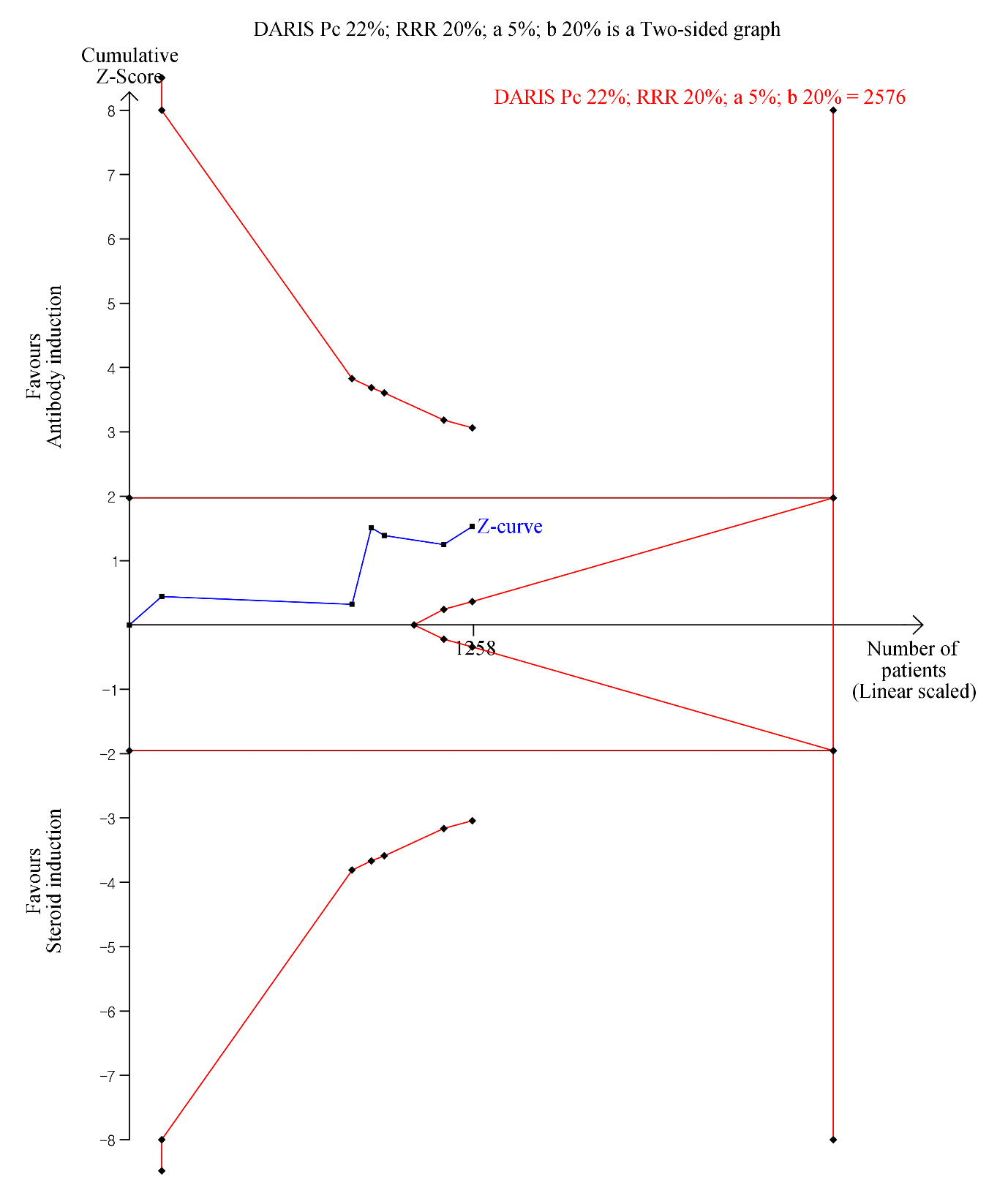


**Supplemental Figure S8.** Trial sequential analysis for HCV recurrence.


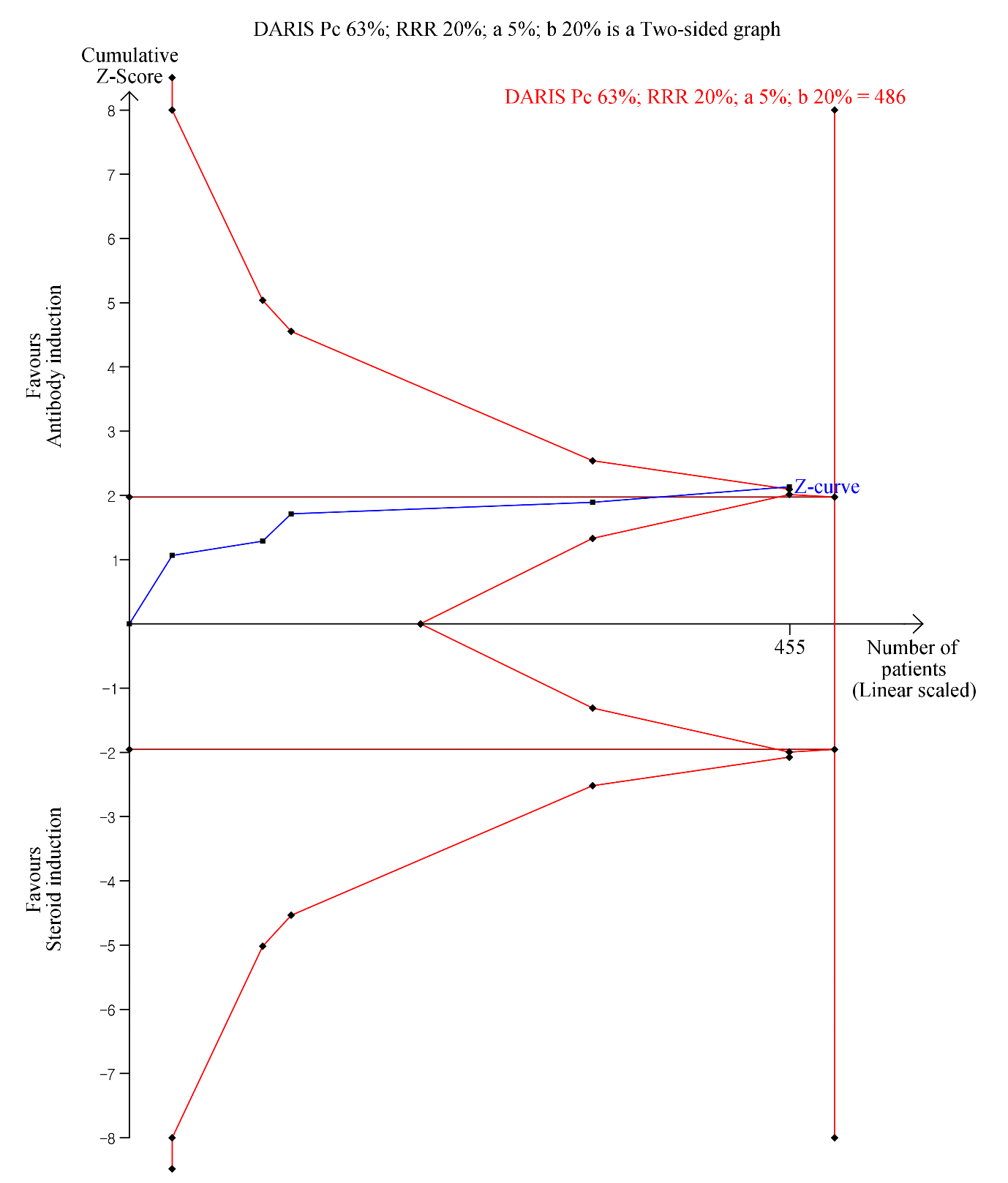


**Supplemental Figure S9.** Trial sequential analysis for hypertension.


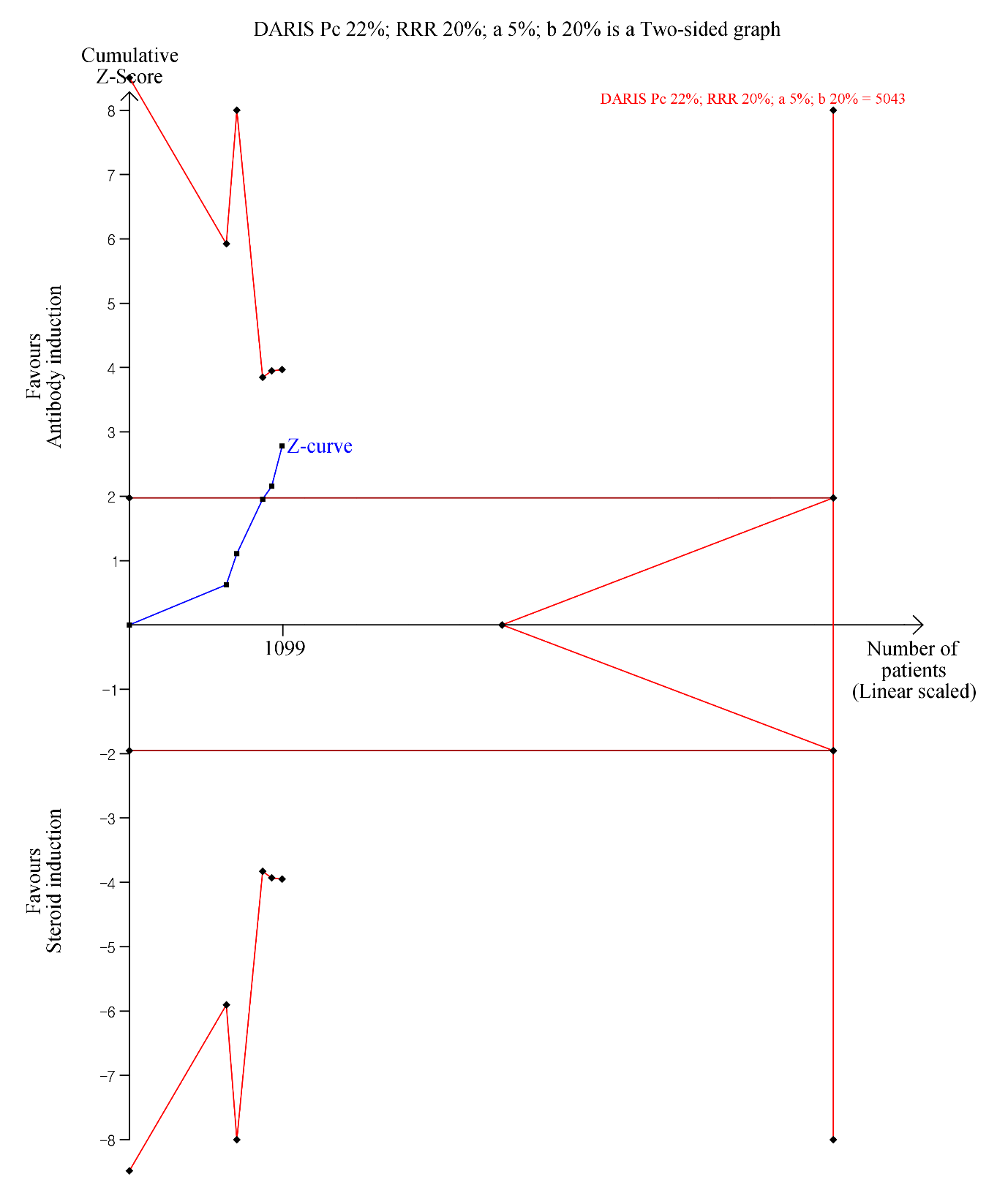


**Supplemental Figure S10.** Results of meta-regression analyses showing how the primary outcome is not influenced by the differences in recipient age, sex ratio, laboratory MELD score, and cold ischemia time. Each circle represents an individual study, and the solid line represents the regression line.

A B


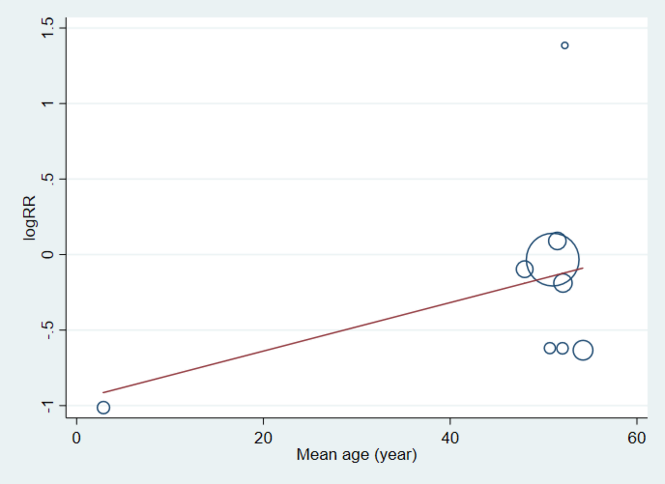

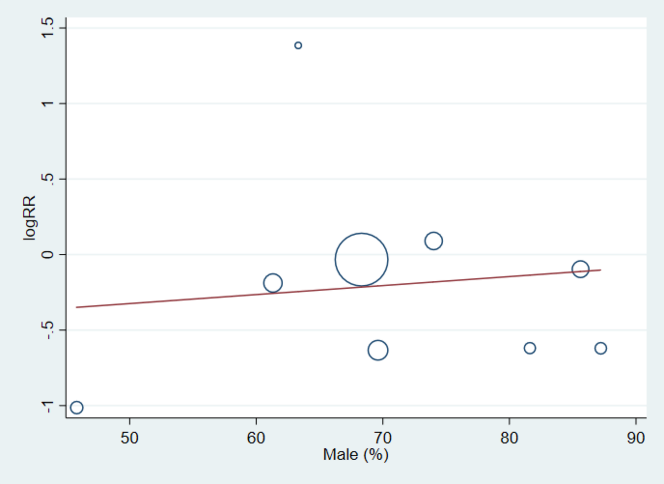


C D


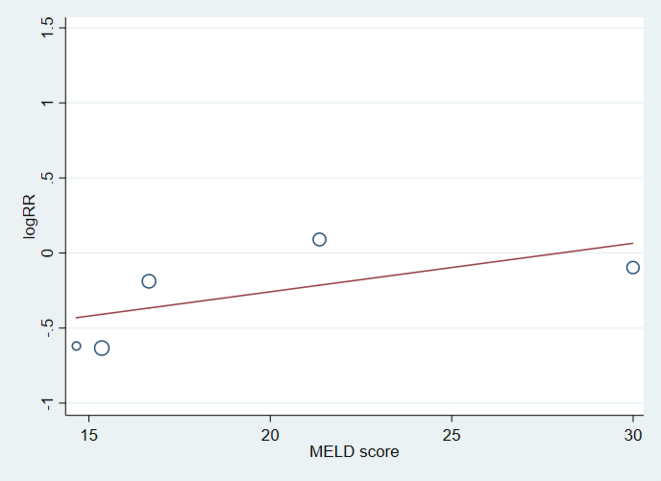

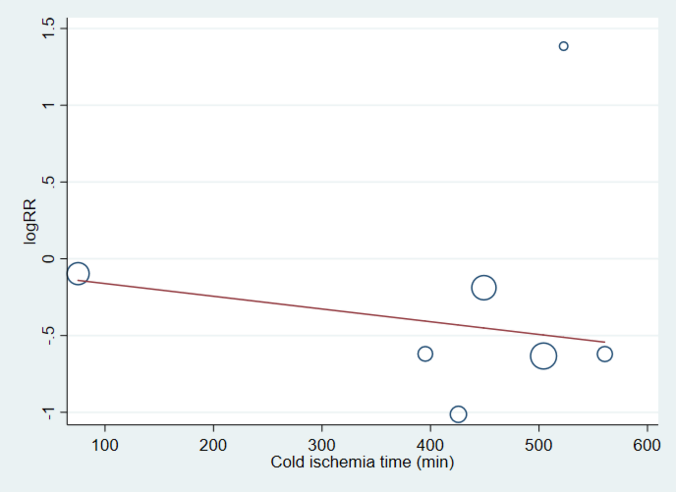


A: mean age (R2=54.51%, P=0.199), B=Sex ratio (male%, R2=-46.70%, P=0.696), C=laboratory MELD score (R2=0.00%, P=0.375), D=cold ischemia time (R2=0.00%, P=0.478).

**Supplemental Table S1.** Distribution of study outcomes across the included trials.

| Outcomes | Boillot  2005 ^5^ | De Simone  2007 ^19^ | Eason  2003 ^26^ | Kathirvel  2021 ^4^ | Kato  2001 ^20^ | Kato  2007 ^20^ | Klintmalm  2011 ^25^ | Lupo  2008 ^24^ | Neumann  2012 ^23^ | Spada  2006 ^22^ | Washburn  2001 ^21^ |
| --- | --- | --- | --- | --- | --- | --- | --- | --- | --- | --- | --- |
| Mortality | ✓ | ✓ | ✓ | ✓ |  | ✓ | ✓ | ✓ | ✓ | ✓ | ✓ |
| Graft loss | ✓ | ✓ | ✓ | ✓ |  | ✓ | ✓ | ✓ | ✓ | ✓ | ✓ |
| Acute rejection | ✓ | ✓ | ✓ | ✓ | ✓ | ✓ | ✓ | ✓ | ✓ | ✓ | ✓ |
| Acute rejection requiring treatment | ✓ |  |  | ✓ |  |  | ✓ | ✓ | ✓ | ✓ | ✓ |
| Corticosteroid resistant rejection | ✓ |  |  | ✓ |  |  |  |  | ✓ | ✓ |  |
| Adverse events | ✓ |  |  |  |  | ✓ |  | ✓ | ✓ |  |  |
| Infection | ✓ |  | ✓ | ✓ |  |  | ✓ | ✓ |  | ✓ |  |
| CMV infection | ✓ |  | ✓ | ✓ |  |  | ✓ | ✓ |  | ✓ |  |
| HCV recurrence |  |  | ✓ |  |  |  | ✓ | ✓ | ✓ | ✓ | ✓ |
| Malignancy | ✓ |  |  |  |  |  | ✓ | ✓ | ✓ | ✓ | ✓ |
| PTLD | ✓ |  |  |  |  |  |  | ✓ | ✓ | ✓ | ✓ |
| Total length of hospital stay |  |  |  | ✓ |  |  |  | ✓ |  |  |  |
| Renal failure requiring dialysis | ✓ |  |  | ✓ |  |  |  |  |  | ✓ |  |
| GFR |  |  |  | ✓ |  |  |  |  | ✓ | ✓ |  |
| Serum creatinine | ✓ |  |  |  |  |  | ✓ |  | ✓ |  |  |
| Diabetes mellitus | ✓ | ✓ | ✓ | ✓ |  | ✓ | ✓ | ✓ | ✓ | ✓ | ✓ |
| Hyperlipidemia | ✓ | ✓ |  | ✓ |  |  | ✓ |  |  | ✓ |  |
| Serum cholesterol | ✓ |  |  | ✓ |  |  |  |  |  | ✓ | ✓ |
| Hypertension | ✓ | ✓ |  | ✓ |  | ✓ |  |  |  | ✓ | ✓ |

CMV = cytomegalovirus; HCV = hepatitis C virus; PTLD = post-transplant lymphoproliferative disorder; GFR = glomerular filtration rate.

**Supplemental Table S2**. Results of the sensitivity analyses using a random-effects model.

| Outcomes | Studies | Antibody | Steroid | Effect size [95% CI]* | P-value | I^2^ |
| --- | --- | --- | --- | --- | --- | --- |
| Mortality ^4,5,19-26^ | 10 | 879 | 804 | 0.90 [0.63, 1.28] | 0.56 | 29% |
| Graft failure ^4,5,19-26^ | 10 | 879 | 804 | 1.03 [0.77, 1.39] | 0.82 | 24% |
| Acute rejection ^4,5,19-26^ | 11 | 879 | 804 | 0.86 [0.72, 1.01] | 0.07 | 0% |
| Acute rejection requiring treatment ^4,5,21-25^ | 7 | 693 | 611 | 0.91 [0.73, 1.12] | 0.36 | 0% |
| Corticosteroid resistant rejection ^4,5,22,23^ | 4 | 506 | 503 | 0.89 [0.29, 2.77] | 0.84 | 45% |
| Adverse events ^4,5,22,23^ | 4 | 475 | 475 | 0.96 [0.85, 1.09] | 0.54 | 76% |
| Infection ^4,5,22,24-26^ | 6 | 671 | 587 | 0.86 [0.70, 1.05] | 0.13 | 0% |
| CMV infection ^5,22,24-26^ | 5 | 619 | 535 | 0.50 [0.33, 0.75] | 0.0008 | 3% |
| HCV recurrence ^21-26^ | 6 | 298 | 229 | 0.90 [0.81, 1.00] | 0.05 | 0% |
| Malignancy ^5,21-25^ | 6 | 590 | 582 | 0.80 [0.32, 2.00] | 0.63 | 0% |
| PTLD ^5,21-24^ | 5 | 485 | 487 | 1.00 [0.07, 15.38] | 1.00 | N/A |
| Total length of hospital stay (days) ^4,25^ | 2 | 78 | 73 | 0.19 [-3.26, 3.64] | 0.91 | 0% |
| Renal failure requiring dialysis ^4,5,22^ | 3 | 439 | 435 | 1.23 [0.55, 2.88] | 0.58 | 0% |
| GFR ^4,22,23^ | 3 | 118 | 144 | 4.62 [0.83, 8.41] | 0.02 | 3% |
| Serum creatinine level (mmol/L) ^5,23,25^ | 3 | 508 | 466 | 13.93 [7.83, 20.02] | <0.0001 | 0% |
| Diabetes mellitus ^4,5,19-26^ | 10 | 857 | 775 | 0.44 [0.33, 0.57] | <0.0001 | 0% |
| Hyperlipidemia ^4,5,19,22,25^ | 5 | 644 | 566 | 0.77 [0.35, 1.68] | 0.51 | 70% |
| Serum cholesterol level (mg/dL) ^4,5,21,22^ | 4 | 418 | 414 | -16.97 [-29.82, -4.12] | 0.01 | 79% |
| Hypertension ^4,5,19-22^ | 6 | 566 | 563 | 0.68 [0.48, 0.96] | 0.03 | 35% |

*The data are presented as mean difference or risk ratio with its 95% confidence interval (CI).

CMV = cytomegalovirus; HCV = hepatitis C virus; PTLD = post-transplant lymphoproliferative disorder; GFR = glomerular filtration rate; N/A = not available.

**Supplemental Table S3**. Quality of the evidence (GRADE approach).

| Outcomes | No. of studies | No. of Antibody group | No. of Steroid group | Quality assessment | | | | | Quality of evidence |
| --- | --- | --- | --- | --- | --- | --- | --- | --- | --- |
|  |  |  |  | Risk of bias | Inconsistency | Indirectness | Imprecision | Publication bias |  |
| Mortality | 10 | 879 | 804 | Serious ^a^ | Not serious | Not serious | Serious ^d^ | Unlikely | ⊕⊕⊝⊝ Low |
| Graft loss | 10 | 879 | 804 | Serious ^a^ | Not serious | Not serious | Serious ^d^ | Unlikely | ⊕⊕⊝⊝ Low |
| Acute rejection | 11 | 879 | 804 | Serious ^a^ | Not serious | Not serious | Serious ^d^ | Unlikely | ⊕⊕⊝⊝ Low |
| Acute rejection requiring treatment | 7 | 693 | 611 | Serious ^a^ | Not serious | Not serious | Serious ^d^ | Unlikely | ⊕⊕⊝⊝ Low |
| Corticosteroid resistant rejection | 4 | 506 | 503 | Serious ^a^ | Not serious | Not serious | Serious ^d^ | Unlikely | ⊕⊕⊝⊝ Low |
| Adverse events | 4 | 475 | 475 | Serious ^a^ | Serious ^b^ | Not serious | Not Serious | Unlikely | ⊕⊕⊝⊝ Low |
| Infection | 6 | 671 | 587 | Serious ^a^ | Not serious | Not serious | Serious ^d^ | Unlikely | ⊕⊕⊝⊝ Low |
| CMV infection | 5 | 619 | 535 | Serious ^a^ | Not serious | Not serious | Not Serious | Unlikely | ⊕⊕⊕⊝ Moderate |
| HCV recurrence | 6 | 298 | 229 | Serious ^a^ | Not serious | Not serious | Not Serious | Unlikely | ⊕⊕⊕⊝ Moderate |
| Malignancy | 6 | 590 | 582 | Serious ^a^ | Not serious | Not serious | Serious ^d^ | Unlikely | ⊕⊕⊝⊝ Low |
| PTLD | 5 | 485 | 487 | Serious ^a^ | Not serious | Not serious | Serious ^d^ | Unlikely | ⊕⊕⊝⊝ Low |
| Total length of hospital stay | 2 | 78 | 73 | Serious ^a^ | Not serious | Not serious | Serious ^d^ | Likely ^f^ | ⊕⊝⊝⊝ very low |
| Renal failure requiring dialysis | 3 | 439 | 435 | Serious ^a^ | Not serious | Not serious | Serious ^d^ | Likely ^f^ | ⊕⊝⊝⊝ very low |
| GFR | 3 | 118 | 144 | Serious ^a^ | Not serious | Not serious | Serious ^e^ | Likely ^f^ | ⊕⊝⊝⊝ very low |
| Serum creatinine | 3 | 508 | 466 | Serious ^a^ | Not serious | Not serious | Serious ^e^ | Likely ^f^ | ⊕⊝⊝⊝ very low |
| Diabetes mellitus | 10 | 857 | 775 | Serious ^a^ | Not serious | Not serious | Not Serious | Unlikely | ⊕⊕⊕⊝ Moderate |
| Hyperlipidemia | 5 | 644 | 566 | Serious ^a^ | Serious ^b^ | Not serious | Serious ^d^ | Unlikely | ⊕⊝⊝⊝ very low |
| Serum cholesterol | 4 | 418 | 414 | Serious ^a^ | Moderate ^c^ | Not serious | Serious ^e^ | Unlikely | ⊕⊕⊝⊝ Low |
| Hypertension | 6 | 566 | 563 | Serious ^a^ | Not serious | Not serious | Not Serious | Unlikely | ⊕⊕⊕⊝ Moderate |

CMV=cytomegalovirus, HCV=hepatitis C virus, PTLD=post-transplant lymphoproliferative disorder, GFR=glomerular filtration rate

The basis for the assumed risk is provided in the footnotes.

^a^ In most studies, blinding was not performed for participants, personnel, and outcome assessors. Final decision to rate down quality of evidence by one level for risk of bias.

^b^ I^2^ was above 50% with wide variance of point estimates across studies. Final decision to rate down quality of evidence by one level for serious inconsistency.

^c^ Even though the I^2^ was above 50%, the point estimates did not vary widely between studies. Final decision to not rate down quality of evidence for moderate inconsistency.

^d^ Confidence interval included null effect as well as appreciable benefit and/or harm. Final decision to rate down quality of evidence by

one level for serious imprecision.

^e^ Optimal information size not reached. Final decision to rate down quality of evidence by one level for serious imprecision.

^f^ The number of studies were less than 4. Final decision to rate down quality of evidence by one level for likely publication bias.

High quality means that we are very confident that the true effect lies close to that of the estimate of the effect.

Moderate quality means that we are moderately confident in the effect estimate: The true effect is likely to be close to the estimate of the effect, but there is a possibility that it is substantially different.

Low quality means that our confidence in the effect estimate is limited: The true effect may be substantially different from the estimate of the effect.

Very low quality means that we have very little confidence in the effect estimate: The true effect is likely to be substantially different from the estimate of effect.
